# Mechanical ventilation-associated pneumonia caused by *Acinetobacter baumannii* in Northeast China region: analysis of genotype and drug resistance of bacteria and patients’ clinical features over 7 years

**DOI:** 10.1186/s13756-021-01005-7

**Published:** 2021-09-15

**Authors:** Tao Zhang, Xiao Xu, Cai-Fang Xu, Salisu Rabiu Bilya, Wei Xu

**Affiliations:** Department of Pediatrics, Shengjing Hospital of China Medical University, No. 36, SanHao Street, Shenyang City, 110004 Liaoning Province People’s Republic of China

**Keywords:** *Acinetobacter baumannii*, Children, Carbapenem, Drug resistance gene

## Abstract

**Objective:**

To investigate the clinical features and outcomes of patients with mechanical ventilation-associated pneumonia (VAP) caused by *Acinetobacter baumannii* (Ab), and to characterize the drug resistance of pathogenic strains and carbapenem resistance-associated genes.

**Methods:**

Clinical data were collected from the PICU of Shengjing Hospital. Patients who met the diagnostic criteria of VAP and for whom Ab was a pathogen were selected as study participants. The patients were divided into carbapenem-resistant *A. baumannii* (CRAB) and carbapenem-sensitive *A. baumannii* (CSAB) groups. The genes closely associated with Ab resistance to carbapenems and the efflux pump-related genes were detected by real-time polymerase chain reaction, and results compared between the two groups.

**Results:**

The total mechanical ventilation time and the administration time of antibiotics after a diagnosis of Ab infection were significantly higher in the CRAB group. And the CRAB group strains were only sensitive to amikacin, cephazolin, compound sulfamethoxazole, and tigecycline. Genetic test results indicated that *IPM* expression was not significantly different between two groups. The *OXA-51* and *OXA-23* in the CRAB group was markedly higher than that in the CSAB group, while *OXA-24* expression was markedly lower. The expression of *AdeABC* and *AdeFGH* was significantly greater in the CRAB compared to CSAB group.

**Conclusion:**

In pediatric patients with VAP caused by Ab infection, the detection rate of CRAB strains is far higher than that of CSAB strains; The abnormal expression of β-lactamase-producing genes (*OXA-23, OXA-24*, and *OXA-51*) and efflux pump-related genes (*AdeABC* and *AdeFGH*) is closely related to the production of CRAB.

## Introduction

*Acinetobacter baumannii* (Ab) is a type of non-sugar-fermenting Gram negative bacillus. It can adapt to the environment and is a common pathogenic bacterium in hospital-acquired infections. *A. baumannii* infection usually occurs in critical patients within the intensive care unit (ICU), where it may induce mechanical ventilation-associated pneumonia (VAP), and infections of wounds, the bloodstream, urinary tract, and other organs, as well as secondary meningitis [1, 2]. As reported by China’s antimicrobial resistance surveillance system in 2019, Ab was ranked No. 4 among clinically isolated Gram negative strains and accounted for 9.7% of all isolated Gram negative strains. Since critical pediatric patients often have immune deficiency and severe underlying disease, and require long-time hospitalization and invasive procedures, the incidence rate of Ab infection and the drug resistance rate of Ab are generally higher in a pediatric intensive care unit (PICU) than in other wards [3, 4]. *A. baumannii* has become a major conditioned pathogenic bacterium of VAP in several PICUs and accounts for 34.7–47.0% of isolated strains [5, 6].

With good efficacy and relatively high safety, carbapenems are usually used as first-line drugs for hospital-acquired Ab infection in adults and children. Although many factors contribute to Ab drug resistance, the abuse of the above antibiotics in either adults or children is an independent risk factor for drug-resistant Ab strains [3]. In 2019, the detection rate of carbapenem-resistant *A. baumannii* (CRAB) strains (mostly extensive drug resistance [XDR] strains) was up to 56% in China. Similarly, the isolation rate of CRAB strains is also greater than 50% in European and American hospitals [7]. The susceptibility of hospital patients to Ab and the high incidence rate of CRAB can significantly increase the death rate, prolong the length of hospital stay, and increase treatment costs of infected patients. This problem has thus attracted extensive worldwide concerns [8] and has promoted numerous investigations on the mechanism of Ab drug resistance. A recent study [9] has suggested that Ab exhibits several drug resistance mechanisms, including β-lactamase production, aminoglycoside-modifying enzyme production, efflux pump, permeability defects, and target modification; a mutation of corresponding genes occurs in these different mechanisms. According to several Chinese studies as well as those in other countries [10–14], an outbreak of hospital-acquired Ab infection is often accompanied by a mutation in strain genotypes (e.g., *OXA-23, OXA-24, OXA-51, OXA-58*, etc.). The high expression of several efflux pump-related genes (e.g., *Ade* series) can also result in Ab resistance to multiple antibiotics (including carbapenems) [15]. In addition, in a study (2016) involving 40 patients requiring long-term hospitalization to investigate changes in the gene profile of Ab strains within a large time span of 829 days, it was found that Ab strains not only increased the expression of some drug resistance genes by overexpressing pmrAB and adeRS transcripts, but can also rapidly acquire drug resistance by recombining exogenous genes. This new perspective explains why Ab strains have a high-drug resistance rate [16].

Due to epidemiological differences, Ab strains isolated clinically show varying genotypes in different countries and regions [15]. For example, in 2020, a dominant multi-drug-resistant Ab strain with *OXA-24* and *OXA-23* mutations was reported in Albania [17]. In 2012, an outbreak in Brazil involved a hospital-acquired Ab infection with an *OXA-58* mutation [11]. In 2011, a reported epidemic of an Ab strain with a *OXA-24* mutation occurred in Spain [18]. In Teheran, Iran, drug-resistant Ab strains were mainly associated with *OXA-24* and *OXA-58* mutations [14]. Ab strains reported in South and Northwest China also had different genotypes [10, 12], and patients infected by Ab strains of different genotypes have demonstrated a difference in clinical manifestations, antibiotic resistance, and treatment outcomes. Considering the high risk of Ab infection, high incidence rate of CRAB, and high death rate in critical patients of the PICU, it is necessary to enhance the monitoring of hospital-acquired Ab infections in various regions. This is to increase our understanding of the drug resistance mechanism and changes in Ab strains so as to provide guidance for clinical treatments, and to offer data support in the research and development of new drugs.

The PICU in Shengjing Hospital of China Medical University is a regional treatment center for critical pediatric patients in Liaoning, Jilin, and Heilongjiang provinces of Northeast China. Thus, its detection profile of Ab strains likely reflects the epidemiological characteristics of Ab in Northeast China. Since patients on mechanical ventilation have a high risk of nosocomial infection in the PICU, we retrospectively collected the data of patients with VAP caused by hospital-acquired Ab infection in the past seven years. Patients were divided into a CRAB group and a carbapenem-sensitive *A. baumannii* (CSAB) group according to drug resistance test results of Ab. Ab strains were recovered, and drug resistance tests were performed as well as the detection of key drug resistance genes. This study aimed to systematically investigate the epidemiological characteristics and drug resistance mechanisms of Ab in the PICU of Northeast China, and to provide new inspiration and suggestions for the clinically targeted detection of drug resistance genes, and the early diagnosis and treatment of CRAB infections.

## Materials and methods

### Collection of clinical data

The study was approved by the Ethics Committee of Shengjing Hospital of China Medical University (2019PS431k). Clinical data were collected from all patients in the PICU of Shengjing Hospital of China Medical University diagnosed with Ab according to clinical isolation and culture from January 1, 2012 to December 31, 2018. Patients with VAP caused by hospital-acquired Ab infection were selected according to the following inclusion and exclusion criteria, and their clinical data were analyzed.

Inclusion criteria: (1) 28 days < age ≤ 14 years; (2) patients on invasive mechanical ventilation; (3) mechanical ventilation time > 48 h with a positive Ab culture; (4) if the sample was from deep airway sputum or bronchoalveolar lavage fluid (BALF), the colony count complied with criteria (> 10^5^ colony forming units [CFU]/mL for endotracheal aspirate and > 10^4^ CFU/mL for BALF); and (5) patients who met the diagnostic criteria of VAP [19].

Exclusion criteria: (1) patients who were hospitalized or underwent surgery in the previous month, or who required hemodialysis or long-term ICU care and intravenous catheterization or other transcutaneous catheterization; (2) patients who were found with other possible pathogenic strains beyond Ab by sample culture (excluding patients whose clinical symptoms and laboratory test results improved and clinical symptoms alleviated within the expected time after antibiotic treatment not targeting Ab infections); and (3) patients with non-eligible sputum samples (the sputum samples failed to meet the criteria of < 10 squamous epithelial cells and > 25 white blood cells in each low-power field).

The following clinical data were collected: age, sex, primary disease, underlying disease, Pediatric Logistic Organ Dysfunction 2 (PELOD2) score at admission, length of hospital stay, drug resistance test results of Ab strains, administration of antibiotics, and prognosis.

### Drug resistance test of Ab strains and identification of CRAB strains

*A. baumannii* strains meeting the criteria in “Collection of clinical data” section were extracted from the microbial specimen bank of our hospital, and then prepared as a bacterial suspension after culturing. Thereafter, drug sensitivity was analyzed using a VITEK-2 compact automatic microbial analyzer (BioMérieux, Marcy l’Etoile, France) and E-test method. Drug sensitivity tests were conducted strictly according to the method recommended by the American Clinical and Laboratory Standard Institute (2015). *Escherichia coli* (ATCC25922) and *Pseudomonas aeruginosa* (ATCC27853) were used as quality control strains. The test results were evaluated at three levels: sensitive, medium, and resistant.

Meropenem and Imipenem were selected as test drugs to evaluate carbapenem resistance; in the case of a medium or resistant test result [20, 21], this indicated a CRAB strain; for two sensitive test results, this indicated a CSAB strain.

### Selection of drug resistance genes of Ab strains

Based on the available literature [15, 16, 22], we selected key genes related to the resistance of Ab to carbapenems for real-time polymerase chain reactions (RT-PCR), including metal-β-lactamase (MBL)-producing genes (*IPM* and *VIM*), hydrolytic-β-lactamase (*CHDL*)-producing genes (*OXA*-*58*, *OXA-51, OXA-23,* and *OXA-24*), and efflux pump-related genes (*AdeA, AdeB, AdeC, AdeF, AdeG,* and *AdeH*).

### RT-PCR detection procedures

After culturing, Ab strains were prepared as a bacterial suspension, and then RNA extracted using Trizol, chloroform, and dehydrated alcohol. Subsequently, residual DNA in samples was removed using RNAiso TM Plus (Takara, Tokyo, Japan) and PrimeScript RT (Takara) kits. Copy DNA was synthesized by the reverse transcription of RNA. Finally, RT-PCR detection was performed with a real-time quantitative PCR kit (Takara). The detection results were corrected using a house-keeping gene (*16 s rRNA*, ATCC19606), and then the relative expression levels of target genes in the samples were calculated for each group. The sequences of primers are shown in Table 1.

**Table 1:** Primer sequences of each gene

Gene	Sequence
IPM-F	GTTACGGTGAAAATCCACACC
IPM-R	AATGATTTCACGCACTCAAGG
VIM-F	GCATTCGACCGACAACTTAGT
VIM-R	TCCGGGTAGTGTTGTTGAATC
OXA23-F	TCCCAGTCTATCAGGAACTTGC
OXA23-R	GGCGTAACCTTTAATGGTCCTA
OXA24-F	AGACGGACTGGCCTAGAGCTA
OXA24-R	ATCGGTTATGTGCAAGGTCATC
OXA51-F	TCCAACAAGGCCAAACTCAAC
OXA51-R	CTTCTGTGGTGGTTGCCTTATG
OXA58-F	CAAATAGGCACGGAAGTTGAT
OXA58-R	CACTTGTTGCTGAACTTCAGGT
AdeA-F	CAATATCGTCAGGCTCTAGCC
AdeA-R	GACCAATGCACCTTCAGTGAC
AdeB-F	GTAACGCCACAATGGAATAAGG
AdeB-R	AATCAGTTGTTCCATTTCACGC
AdeC-F	TGCAGTTTCAGGATTAGACCTT
AdeC-R	TACTGGCTCATGCAATAACACA
AdeF-F	ATATGGGACTTGCCAATGAAAC
AdeF-R	ATTCGTGCATATAGGCCTGGTA
AdeG-F	TAACTCGCTTACCCTATCTCCTG
AdeG-R	CTTTACCATAGTTGTCTGACGCA
AdeH-F	TATTAGGAAAACCACCAGCAGAC
AdeH-R	ATATCGGGTCGTCTTTCAAGTAA
16 s rRNA-F	GTAGCTTGCTACTGGACCTAG
16 s rRNA-R	CATACTCTAGCTCACCAGTATCG

### Statistical analysis

Quantitative data of normal distribution were presented as mean ± standard deviation (SD), while those of abnormal distribution were expressed as the median and quartile; the quantitative data of normal distribution, with or without homogeneity of variance, were compared between two groups using a *t* test or rank sum test. Qualitative data were presented as a percentage (%) and their inter-group comparison was performed with a *x*^2^ test; qualitative data incapable of being analyzed with a *x*^2^ test were compared between two groups using Fisher’s exact test. SPSS 17.0 software was used for statistical analysis, and *P* < 0.05 indicated a statistically significant difference.

## Results

### General data

In total, 541 cases of Ab-positive samples were collected during 2012 and 2018. According to inclusion and exclusion criteria (the positive samples in the first culture were included in the study for patients with Ab-positive repeated cultures), 105 patients (including 58 males) with VAP caused by hospital-acquired Ab infection were eligible for this study. These patients had a median age of 27 months, of which 77.1% were admitted to hospital for infectious disease and nearly 50% had underlying disease; Ab strain samples were mainly collected from the sputum and accounted for 75% of all samples; the median total mechanical ventilation time, total administration time of antibiotics, and total length of hospital stay was 94 h, 22 days, and 39 days, respectively; 29/105 (27.6%) of cases died.

**Fig. 1:**
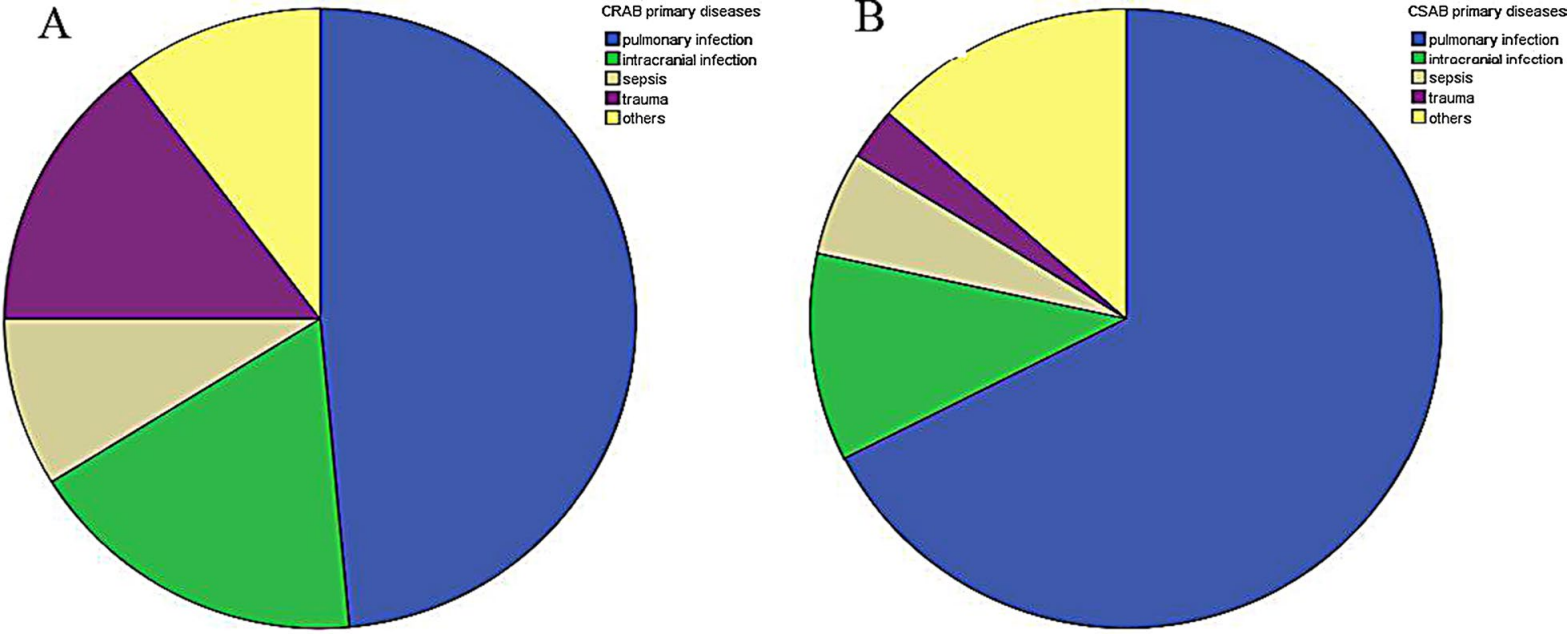
Classification of primary diseases in each group. **A**: CRAB group, **B**: CSAB group

Based on drug resistance test results, patients were divided into CRAB (n = 68, 64.8%; 39 males, 57.3%) and CSAB (n = 37, 35.2%; 19 males, 51.4%) groups. For CRAB and CSAB groups, the median age was 32 and 24 months, the median length of hospital stay was 41 and 35 days, the primary disease was infectious disease in 51 and 30 cases, 33 and 19 cases had underlying disease, and 20/68 (29.4%) and 9/37 (24.3%) of cases died, respectively. In the general data (including age, PELOD2 score at admission, percentage of patients with infectious disease as primary disease, and percentage of patients with underlying disease), no statistically significant difference was found between the two groups. In respect of treatment and prognosis, no statistically significant difference was found in the total length of hospital stay and death rate between the two groups. However, the total mechanical ventilation time in the CRAB group was significantly longer than that in the CSAB group. Meanwhile, the administration time for antibiotics after a diagnosis of VAP caused by Ab infection was markedly longer in the CRAB than CSAB group, though there was no statistical difference in the total administration time of antibiotics between the two groups. The details are shown in Table 2 and Fig. 1.

**Table 2:** General information statistics

	Total (105)	CRAB (68)	CSAB (37)	
Gender (male,%)	58 (55.2)	39 (57.3)	19 (51.4)	
Age (m)	27 (6, 60)	32 (7, 60)	24 (4, 72)	*P* = 0.497
*Specimen source (%)*				
Sputum	75 (71.4)	47 (69.1)	28 (75.7)	
Endotracheal tube	16 (15.2)	14 (20.5)	2 (5.4)	
Alveolar lavage fluid	7 (6.7)	5 (7.3)	2 (5.4)	
Others	7 (6.7)	2 (2.8)	5 (13.5)	
PELOD2 score (Admitted within 24 h), M (IQR)	2 (0, 5)	3 (0, 5)	2 (1, 3)	*P* = 0.17
*Primary disease was infectious or not*				
Yes	81 (77.1)	51 (75.0)	30 (81.1)	*P* = 0.628
No	24 (22.9)	17 (25.0)	7 (18.9)	
*Classification of primary disease (%)*				
Pulmonary infection	58 (55.2)	33 (48.5)	25 (67.6)	
Intracranial infection	16 (15.2)	12 (17.6)	4 (10.8)	
Sepsis	8 (7.6)	6 (8.8)	2 (5.4)	
Trauma	11 (10.5)	10 (14.7)	1 (2.7)	
Others	12 (11.4)	7 (10.3)	5 (13.5)	
*Underlying disease*				
Yes	52 (49.5)	33 (48.5)	19 (51.4)	*P* = 0.84
No	53 (50.5)	35 (51.5)	18 (48.6)	
*Classification of underlying diseases (%)*				
Heart diseases	18 (17.1)	12 (17.6)	6 (16.2)	
Muscle diseases	12 (11.4)	6 (8.8)	6 (16.2)	
Metabolic diseases	5 (4.8)	5 (7.4)	0	
Respiratory malformation	5 (4.8)	4 (5.9)	1 (2.7)	
Blood diseases and tumors	5 (4.8)	3 (4.4)	2 (5.4)	
Multiple malformations	5 (4.8)	4 (5.9)	1 (2.7)	
*Other invasive operations (%)*				
Deep vein catheterization	40 (38.1)	34 (50)	6 (16.2)	
Bronchonscope	18 (17.1)	14 (20.6)	4 (10.8)	
CRRT	11 (10.5)	7 (10.3)	4 (10.8)	
Thoracocentesis	19 (18.1)	15 (22.1)	4 (10.8)	
Peritonrocentesis	2 (2.0)	2 (2.9)	0	
Catheterization	63 (60.0)	46 (67.6)	17 (45.9)	
Surgery	28 (26.7)	21 (30.9)	7 (18.9)	
Total mechanical ventilation time (h), M (IQR)	94 (1, 270)	215 (119.5, 370.5)	136 (64, 300)	***P* = 0.035**
Total antibiotic use time (d), M (IQR)	22 (18, 31)	22.5 (17, 34)	22 (18, 26)	*P* = 0.207
Total hospital stay (d), M (IQR)	39 (22, 64)	41 (24, 59)	35 (19, 64)	*P* = 0.487
*Major tests within 48 h of Ab positive, M (IQR)*				
WBC (10^9^/L)	10.6 (7.3, 14.6)	9.5 (6.5, 14.4)	11.1 (8.5, 16.7)	*P* = 0.145
CRP (mg/L)	16.8 (6.3, 68.7)	17.8 (7.5, 75.3)	14.2 (5.1, 51.6)	*P* = 0.388
PCT (ng/ml)	1.0 (0.3, 6.2)	1.4 (0.3, 6.2)	0.7 (0.3, 3.4)	*P* = 0.544
Hb (g/L)	92 (82, 105)	92 (82, 104)	91 (83.5, 104)	*P* = 0.817
PLT (10^9^/L)	266.5 (133.5, 430.5)	261 (127, 435)	306 (156.9, 408)	*P* = 0.58
Mechanical ventilation time after Ab positive (h), M (IQR)	94 (24, 245)	62 (23.5, 268)	112 (24, 220)	*P* = 0.936
Antibiotic use time after Ab positive (d), M (IQR)	10 (5, 19)	11.5 (5.5, 23.5)	8 (5, 12)	***P* = 0.043**
Length of hospital stay after Ab positive (d), M (IQR)	21 (10, 39)	20.5 (8.5.39)	26 (13, 38)	*P* = 0.407
*Outcome*				
Survive	78 (74.3)	48 (70.6)	28 (75.7)	*P* = 0.652
Dead	29 (25.7)	20 (29.4)	9 (24.3)	

The bold represent statistically significant

The *p*-value was the result of comparing the CRAB and CSAB group

### Drug resistance statistics

As revealed by drug resistance test results, for 105 cases, Ab strains were generally resistant to the common β-lactamase antibiotics, with or without the addition of enzymes. For instance, of these drugs, a drug resistance rate of > 50% was prevalent for cefepime, ceftazidime, ampicillin, ampicillin sulbactam, as well as piperacillin tazobactam, and even > 80% for ampicillin and cefepime; Ab strains were only sensitive to cephazolin. Ab strains also had a drug resistance rate of > 50% for three quinolinone antibiotics (gemifloxacin, ciprofloxacin, and levofloxacin), but they were sensitive to other types of antibiotics, such as aminoglycosides (e.g., amikacin), sulfonamides (e.g., compound sulfamethoxazole), and tetracyclines (e.g., tigecycline).

**Fig. 2:**
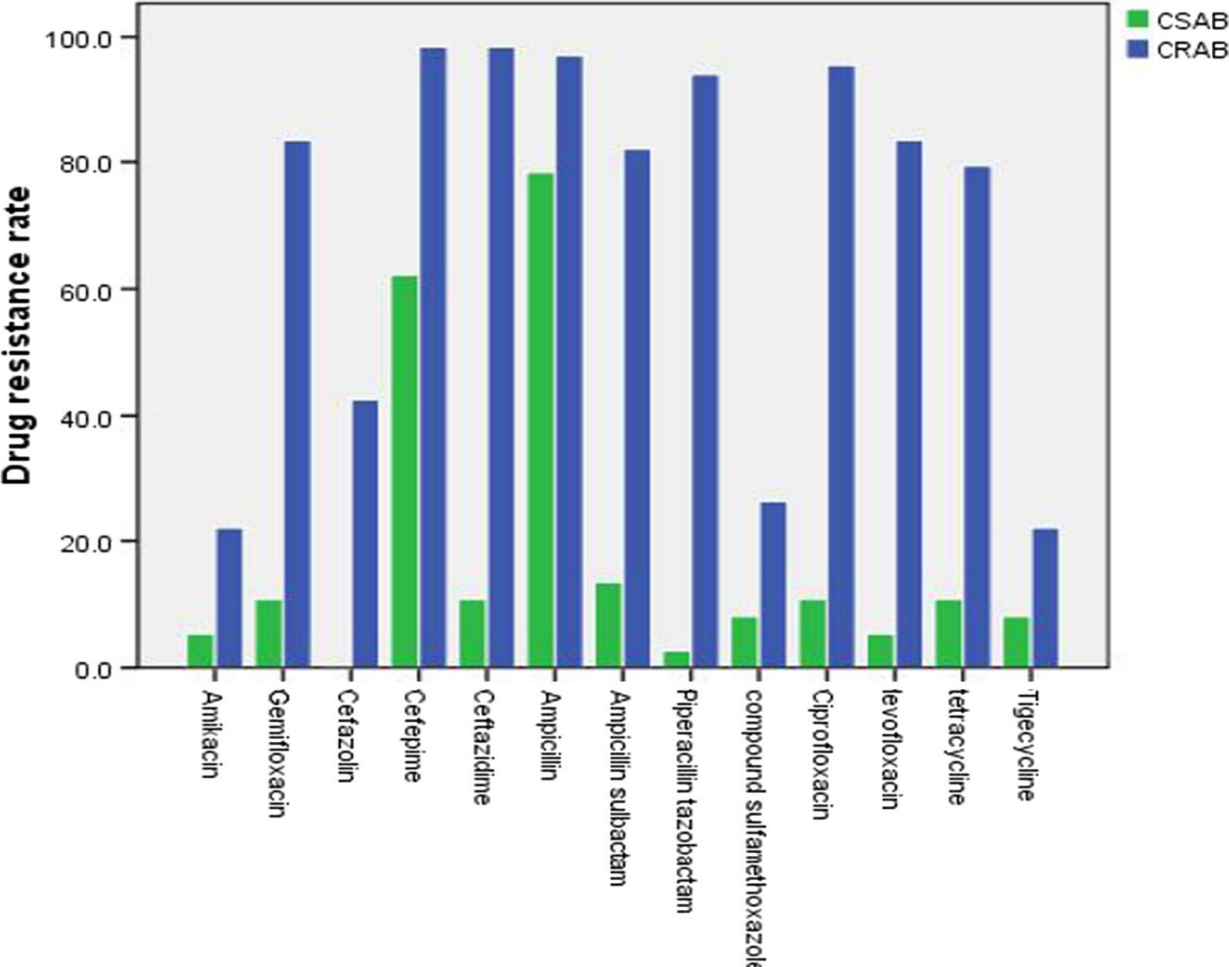
The drug resistance rate compared between CRAB and CSAB group

Based on statistics by group, Ab strains were sensitive to amikacin (drug resistance rate: 22.1%), cephazolin (42.6%), compound sulfamethoxazole (26.5%), and tigecycline (22.1%), but resistant to the remaining nine antibiotics (drug resistance rate: > 50%, or nearly 100% for partial antibiotics [e.g., cefepime, ceftazidime, ampicillin, piperacillin tazobactam, as well as ciprofloxacin]) in the CRAB group. In the CSAB group, Ab strains were sensitive to nearly all the remaining 11 antibiotics (excluding cefepime and ampicillin). The statistical results of Ab antibiotic resistance showed no significant difference in drug resistance between the two groups, but Ab strains were more sensitive to the remaining 12 antibiotics in the CSAB compared to CRAB group. According to the classification of antibiotics and the analysis of the drug resistance of Ab strains in various groups, up to 19 (27.9%) cases of XDR + pan drug resistance (PDR) strains existed in the CRAB group, far higher than that in the CSAB group; the difference between the two groups was statistically significant. Detailed data are shown in Table 3 and Fig. 2.

**Table 3:** The results of drug resistance test

	Total (105)	CRAB (68)	CSAB (37)	
*Amikacin*				
Drug resistance strains/rat (%)	17 (16.2)	15 (22.1)	2 (5.4)	*P* = 0.029
Sensitive strains	88	53	35	
*Gemifloxacin*				
Drug resistance strains/rat (%)	61 (58.1)	57 (83.8)	4 (10.8)	*P* = 0.000
Sensitive strains	44	11	33	
*Cefazolin*				
Drug resistance strains/rat (%)	29 (27.6)	29 (42.6)	0 (0)	*P* = 0.000
Sensitive strains	76	39	37	
*Cefepime*				
Drug resistance strains/rat (%)	90 (85.7)	67 (98.5)	23 (62.2)	*P* = 0.000
Sensitive strains	15	1	14	
*Ceftazidime*				
Drug resistance strains/rat (%)	71 (67.6)	67 (98.5)	4 (10.8)	*P* = 0.000
Sensitive strains	34	1	33	
*Ampicillin*				
Drug resistance strains/rat (%)	95 (90.5)	66 (97.1)	29 (78.4)	*P* = 0.003
Sensitive strains	10	2	8	
*Ampicillin sulbactam*				
Drug resistance strains/rat (%)	61 (58.1)	56 (82.4)	5 (13.5)	*P* = 0.000
Sensitive strains	44	12	32	
*Piperacillin tazobactam*				
Drug resistance strains/rat (%)	65 (61.9)	64 (94.1)	1 (2.7)	*P* = 0.000
Sensitive strains	40	4	36	
*Compound sulfamethoxazole*				
Drug resistance strains/rat (%)	21 (20.0)	18 (26.5)	3 (8.1)	*P* = 0.039
Sensitive strains	84	50	34	
*Ciprofloxacin*				
Drug resistance strains/rat (%)	69 (65.7)	65 (95.6)	4 (10.8)	*P* = 0.000
Sensitive strains	36	3	33	
*Levofloxacin*				
Drug resistance strains/rat (%)	59 (56.2)	57 (83.8)	2 (5.4)	*P* = 0.000
Sensitive strains	46	11	35	
*Tetracycline*				
Drug resistance strains/rat (%)	58 (55.2)	54 (79.4)	4 (10.8)	*P* = 0.000
Sensitive strains	47	14	33	
*Tigecycline*				
Drug resistance strains/rat (%)	18 (17.1)	15 (22.1)	3 (8.1)	*P* = 0.103
Sensitive strains	87	53	34	
*XDR + PDR*				
Yes/rate (%)	20 (19.0)	19 (27.9)	1 (2.7)	*P* = 0.001
No	85	49	36

### Genetic test results

The genetic test results of 105 strains revealed that *VIM* (a MBL-producing gene) and *OXA-58* (a CHDL-producing gene) were only expressed in over 10 Ab strains at an extremely low level; they were not detected in the majority of strains. The expression rate of other genotypes in all Ab strains was > 80%. According to the analysis by group, a significant difference in the expression of *IPM* (a MBL-producing gene) between CRAB and CSAB groups was not apparent. By combining this result with the near lack of VIM expression, it was thereby inferred that the production of CRAB strains had no tight association with the mutation of MBL-producing genes. As for the CHDL-producing OXA gene series, the expression of *OXA-51* and *OXA-23* in Ab strains was significantly higher in the CRAB than CSAB group, especially for *OXA-51*. *OXA-24* expression in the CRAB group was lower than that in the CSAB group. As indicated by the genetic test results of an efflux pump-related Ade gene series, the expression of *AdeA, AdeB, AdeC, AdeF, AdeG*, and *AdeH* in Ab strains was markedly higher in the CRAB than CSAB group in a statistically significant manner. The details are outlined in Table 4.

**Table 4:** Detection results of drug-resistant gene expression rate and expression level of Ab strain

	Total (105)		CRAB (68)		CSAB (37)		Expression quantity comparison
	Expression number	Expression quantity, M (IQR)	Expression number	Expression quantity, M (IQR)	Expression number	Expression quantity, M (IQR)	
IPM	92 (87.6)	0.099 (0.044, 0.305)	60 (88.4)	0.111 (0.038, 0.311)	32 (86.5)	0.072 (0.044, 0.171)	*P* = 0.358
VIM	10 (9.5)	Undetected	8 (11.8)	Undetected	2 (5.4)	Undetected	–
OXA-58	11 (10.5)	Undetected	4 (5.9)	Undetected	7 (18.9)	Undetected	–
OXA-51	92 (87.6)	0.464 (0.069, 1.800)	62 (91.2)	1.256 (0.129, 2.676)	30 (81)	0.137 (0.043, 0.391)	*P* = 0.000
OXA-23	87 (82.9)	0.126 (0, 0.462)	61 (89.7)	0.271 (0.002, 1.113)	26 (70.3)	0.0001 (0, 1.123)	*P* = 0.000
OXA-24	82 (78.1)	0.206 (0.003, 1.741)	51 (75)	0.116 (0.017, 0.768)	31 (83.8)	0.384 (0.118, 102.162)	*P* = 0.035
AdeH	93 (88.6)	0.097 (0.021, 0.440)	62 (91.2)	0.184 (0.033, 0.563)	31 (83.8)	0.046 (0.01, 0.146)	*P* = 0.004
AdeG	92 (87.6)	0.066 (0.017, 0.241)	62 (91.2)	0.108 (0.018, 0.274)	30 (81)	0.027 (0.016, 0.047)	*P* = 0.006
AdeF	91 (86.7)	0.122 (0.018, 0.515)	62 (91.2)	0.202 (0.029, 0.802)	29 (78.4)	0.073 (0.011, 0.156)	*P* = 0.008
AdeC	92 (87.6)	1.790 (0.665, 4.109)	62 (91.2)	2.382 (0.848, 5.852)	30 (81)	1.085 (0.601, 3.193)	*P* = 0.032
AdeB	92 (87.6)	0.209 (0.052, 0.669)	62 (91.2)	0.324 (0.094, 0.838)	30 (81)	0.12 (0.042, 0.245)	*P* = 0.004
AdeA	91 (86.7)	1.021 (0.283, 2.834)	61 (89.7)	1.478 (0.35, 4.25)	30 (81)	0.766 (0.073, 1.335)	*P* = 0.003

## Discussion

Carbapenems were once the first choice of treatment for Ab infection. However, their long-time use might lead to the high expression of efflux pump-related genes in Ab and thus selectively induce the production of CRAB strains [15]. In China, the percentage of CRAB strains in Ab strains that have been clinically isolated and cultured has exceeded 50% for several successive years. More seriously, most CRAB strains are resistant to both penicillin and other β-lactamase antibiotics [23–25]. This, plus the limited medications available for children, means pediatric physicians, particularly those in the PICU, have to deal with a high incidence rate of hospital-acquired Ab infections, which can lead to difficulty in the selection of antibiotics when treating CRAB. This is the major cause for the high death rate, expensive treatment costs, and long-time hospitalization of patients with CRAB infections.

We first completed the statistics for all patients with an Ab-positive culture result in the past seven years. By screening, 105 patients were suspected of having had VAP caused by hospital-acquired Ab infection when aged < 36 months; their primary disease was mostly infectious disease, predominantly pulmonary and intracranial infections, and trauma. The analysis according to group demonstrated that the percentage of patients with diseases derived from pulmonary infection in the CRAB group (< 50%) was markedly lower than that in the CSAB group, while the percentage of patients with diseases derived from intracranial infection and trauma was significantly higher (about 1/3). Based on the statistics of various invasive procedures except mechanical ventilation, the percentage of patients undergoing bronchoscopic procedures in the CRAB group was lower than that in the CSAB group, while the percentage of patients undergoing other invasive procedures (including deep venous catheterization, thoracic puncture, abdominal puncture, urethral catheterization, and surgery) was significantly greater. Thus, it can be seen that the diversity of invasive procedures is a high risk for CRAB infection. In addition, a careful statistical analysis of the influence of primary disease severity, complications, body immunity, different treatment measures (especially invasive procedures) and antibiotics, mechanical ventilation time, and other possible factors on CRAB susceptibility in different patients was not undertaken because of the small sample size used. In addition, this was a retrospective study in which the collection of complete data was difficult, which might have caused a bias in results. In future, we shall pay particular concerns to patients with pulmonary infection, and shall step up our vigilance of patients on long-term mechanical ventilation who have an intracranial infection and trauma to prevent the occurrence of nosocomial infection.

A PELOD2 score is a recognized measure to evaluate disease severity and the prognosis of patients in comprehensive PICUs. The studied patients had a low PELOD2 score (median: 2) within 24 h after admission. The death rate (as calculated with the prediction formula of the death rate) was only 0.3% [26], but was > 25% as shown by our final statistical results. Such a large difference in the death rate may be related to a lack of successive scoring in our study in our study; it also laterally reflects that most patients in our statistics had no severe diseases at admission. However, the death rate greatly increased once these patients developed VAP caused by Ab infection. This is an explanation of why PICUs need to minimize invasive mechanical ventilation time and good airway care to avoid VAP. In the present study, although a statistically significant difference in the length of hospital stay and death rate between two groups was not observed, the mechanical ventilation time and post-diagnosis administration time for antibiotics were markedly longer in the CRAB compared to CSAB group; this indicates that VAP caused by CRAB infection is more difficult to treat than that caused by CSAB infection.

In this study, Ab strains in the CRAB group were resistant to nearly all β-lactamase antibiotics, with and without the addition of enzyme, and were only sensitive to amikacin, cephazolin, compound sulfamethoxazole and tigecycline; nearly 28% were XDR and PDR strains. More seriously, among several antibiotic options, aminoglycosides, sulfonamides, quinolones, and polymyxins are limited or should be carefully used in children. Aminoglycosides have such side effects as ototoxicity, nephrotoxicity, and neuromuscular blockade; their application, especially in infants, may induce unobservable ototoxicity symptoms, and further cause irreversible deaf-mutism if no drug discontinuation or intervention is implemented. Different types of aminoglycosides have a varying incidence rate of ototoxicity; in our study, the ototoxicity incidence rate of amikacin was 1.5% in the drug sensitivity test [27]. Tetracyclines can influence the teeth of infants and children; infants are more susceptible to such an impact, with even the short-term use of such drugs in infants under one years of age showing a high incidence of tooth invasion. In addition, tetracyclines can also induce skeletal deformation, osteosis suppression, infantile skeleton growth inhibition, and transient growth retardation. Therefore, tetracyclines are forbidden in children under eight years of age. It was found in animal experiments that quinolones can induce cartilage disease and articular toxicity in infantile rats and dogs; thus, these drugs are only recommended for patients ≥ 16 years of age as per manufacturer’s instructions. Polymyxins are the last line of defense for Ab infection because they are not only expensive, but also show tubular cytotoxicity that can lead to acute tubular necrosis [28].

Although the use of the above antibiotics is somewhat limited in pediatric departments and may cause various adverse reactions, pediatric clinicians often choose off-label or the combined use of antibiotics to treat CRAB infection after weighing the benefits and risks. This, however, may lead to an adverse effect on the subsequent growth and development of patients and may also result in a medical dispute. Ab strains in the CSAB group were sensitive to nearly all the 11 remaining antibiotics, excluding cefepime and ampicillin, and therefore treatment was less difficult. This might be one of the reasons for the significantly shorter administration time of antibiotics after Ab-positive culture in the CSAB group. Interestingly, all Ab strains in the CSAB group were sensitive to cephazolin as a first-generation cephalosporin, with strains in the CRAB group also having a sensitivity rate of nearly 57.4%. However, the sensitivity rate of strains to cefepime as a fourth-generation cephalosporin was only 37.8% in the CSAB group and nearly 0% in the CRAB group. Reviewing the application of antibiotics in our department and the purchase of antibiotics in our hospital during the past 10 + years, we suspect that the above phenomenon might be associated with an extremely low application rate of cephazolin and the extensive use of cefepime. This suggests that we perhaps will have more antibiotic options to treat CRAB infections with if selectively and protectively using several antibiotics in our daily work.

*A. baumannii*, as a non-fermenting bacterium, has a long survival time in the natural environment, a rapid and diverse capability of acquiring drug resistance, and varying genotypes. It is thus one of the bacteria most difficult to treat in the clinic. Therefore, it is important for the early diagnosis of Ab and its treatment with appropriate antibiotics to understand the drug resistance mechanisms of Ab and Ab genotypes in local regions and hospitals. Based on differences in gene sequence, β-lactamases are classified into four types: A, B, C, and D [29]. Type A β-lactamases are a kind of cephalosporin hydrolase that mainly produce clavulanic acid; type B β-lactamases are MBLs and their catalysis requires zinc or other heavy metals, while the catalysis of types A, C, and D lactamases depends on serine. MBLs with extensive substrates can induce the hydrolysis of almost all β-lactamase antibiotics, including carbapenems, but have no effect on monocyclic β-lactamase antibiotics. Type C β-lactamases are mainly resistant to cephalomycin, penicillin, and cephalosporins. Type D β-lactamases, also known as OXAs, can usually rapidly hydrolyze oxacillin. Currently, over 400 OXA enzymes have been identified, of which most have the capacity to hydrolyze carbapenems. In summary, types B and D β-lactamases serve as one of the major mechanisms for Ab resistance to carbapenems [25].

*IPM* and *VIM* detected in our study are the common genotypes of MBLs [29]. In the present study, the expression rate of *IPM* was > 80% in both CRAB and CSAB groups. However, a significant difference in the expression level of *IPM* between the two groups was not noted. In both groups, however, *VIM* was rarely expressed in Ab strains. Therefore, we presumed that the drug resistance mechanism of CRAB strains in the PICU of our hospital was not tightly related to MBLs. In the *OXA* series, *OXA-23, OXA-24, OXA-51,* and *OXA*-58 are the most common genotypes of Ab, but their expression varies markedly in different countries and regions. For example, in 2012, the high expression of *OXA-58* in clinical Ab strains was reported in South Brazil [11]; in the Guangdong Province of China, *OXA-58* was reported to be highly expressed in Ab strains isolated before 2008, while *OXA-23* was mainly expressed in those isolated after 2009 [10]. In the past seven years, *OXA-58* was rarely expressed in Ab strains from the PICU of our hospital. Similar to the study in Guangdong Province, the high expression of *OXA-23* was also found in MDR-Ab strains from ICUs in Northwest China [12]. Meanwhile, a low expression level of *OXA-23* in CSAB strains was also observed in another study [30]. *OXA-23* had a high expression rate in Ab strains from the PICU of our hospital, with its expression level in the CRAB group significantly higher than that in the CSAB group. The expression trend of *OXA-51* was nearly consistent with that of *OXA-23*, which is similar to recent study results at Chinese and abroad [13, 31]. In contrast, *OXA-24* had a high expression rate in Ab strains from both groups, but its expression level in the CRAB group was lower than that in the CSAB group. This differs from the results of most studies showing that *OXA-24* was highly expressed in MDR-Ab [14, 17, 18, 32]. The thought was that this might be associated with a small sample size, but it might be that the expression trend of *OXA-24* in Ab strains from the PICU of our hospital was indeed different from that of other regions; this needs to be validated in a subsequent larger-sized study.

In addition to types B and D β-lactamases, the efflux pump is one of the major mechanisms for Ab resistance to carbapenems and tigecycline. To date, four efflux pump-related gene superfamilies have been discovered [25, 33, 34]. *AdeA, AdeB, AdeC, AdeF, AdeG*, and *AdeH* all belong to the resistance-node-cleavage superfamily of efflux pump-related genes. *AdeAB*C is mostly associated with aminoglycoside resistance, and can reduce Ab sensitivity to tigecycline and non-fluoroquinolone antibiotics [35–37]. *AdeFGH* is related to tigecycline resistance [38]; meanwhile, a study [39] showed that *AdeFGH* overexpression induced by low-dose antibiotics was helpful for the biofilm production of Ab strains and increased the drug resistance of Ab. Therefore, we focally selected *AdeA, AdeB, AdeC, AdeF, AdeG,* and *AdeH* for genotype testing. Our final results indicated that the abovementioned genes showed high expression in all Ab strains from the PICU of our hospital, with expression levels significantly greater in the CRAB compared to CSAB group. These findings show that the high expression of *AdeABC* and *AdeFGH* may be involved in inducing the production of CRAB strains.

## Conclusion

In patients with VAP caused by Ab infection from the PICU of our hospital, the detection rate of CRAB strains was far higher than that of CSAB strains. Most CRAB strains were of MDR, XDR, and PDR that were sensitive to amikacin, cephazolin, compound sulfamethoxazole, and tigecycline but were extensively resistant to β-lactamase antibiotics, with or without the addition of enzyme. The difficulty of treating patients in the CRAB group was evidently greater than that in the CSAB group; mechanical ventilation time and post-diagnosis administration time of antibiotics were also significantly longer. As found by genotype testing, the abnormal expression of *OXA-23, OXA-24*, and *OXA-51* (β-lactamase-producing genes) as well as *AdeABC* and *AdeFGH* (efflux pump-related genes) was closely associated with the production of CRAB in the PICU of our hospital. Therefore, the dynamic monitoring of the above genes should be increased in order to detect CRAB strains early and to choose appropriate antibiotics for targeted treatment.

## Data Availability

All authors agreed that the data and materials mentioned in this article are true and availability.

## Abbreviations

Ab: *Acinetobacter baumannii*
VAP: Mechanical ventilation-associated pneumonia
PICU: Pediatric intensive care unit
CRAB: Carbapenem-resistant *A. baumannii*
CSAB: Carbapenem-sensitive *A. baumannii*
XDR: Extensive drug resistance
PDR: Pan drug resistance
PELOD2: Pediatric logistic organ dysfunction 2
MBL: Metal-β-lactamase
RT-PCR: Real time polymerase chain reaction
